# Supporting Safe Surgery with Simple, Low-Cost Solutions

**DOI:** 10.1007/s00268-023-06989-4

**Published:** 2023-03-31

**Authors:** David Watters

**Affiliations:** 1 School of Medicine and Health Sciences, Deakin University, Geelong, Australia; 2 University Hospital Geelong, Barwon Health, Geelong, VIC Australia

This issue of the World Journal of Surgery includes a paper on the evaluation of surgical headlights in the Sub-Saharan Africa [1]. Both countries involved in the study (Ethiopia and Liberia) are low-income countries experiencing frequent power outages and unreliable back-up generators, the latter due to fuel shortages or dis-repair. A battery-powered headlight is not only appropriate technology, but represents normal, everyday practice for surgeons even in high-income countries. The provision of headlights, donated by Lifebox, thus potentially offers best practice lighting even in the presence of bright overhead operating room lights and generally adequate lighting when these fail.

When I worked in sub-Saharan Africa, I remember completing an upper abdominal procedure close to the oesophageal hiatus under torchlight. It was challenging to complete the surgery safely with only one beam of light, as it was difficult to keep my head out of the way whilst still visualising the tissues to be mobilised and divided. I was too far into the operation when the lights failed and the back-up generator failed to kick-in, so I could not abandon the procedure. Although the operation ended successfully and the outcome was good, in retrospect I wish I had been prepared with a surgical headlight for added safety margin when the power and lighting failed. Today there are still predictably, unpredictable power outages which compromise surgery due to poor visibility. Even when power failures do not occur most surgeons who use headlights, as I do now in Australia, would attest to their value in improving visibility of the operative field.

Lifebox is a non-profit organisation that was founded in 2011 by some of the same individuals contributing to WHO’s safe surgery saves lives study group, and who were responsible for the Surgical Safety Checklist [2]. They originally focussed on providing pulse oximeters and to date have distributed over 33,000 free pulse oximeters, as well as providing user training in low resource settings, resulting in less oxygen desaturation events, and thus improved perioperative safety [3]. Recently, Lifebox also introduced a Clean Cut multimodal surgical infection prevention programme, reporting that surgical infections were reduced by a third [4]. During the COVID-19 pandemic, Lifebox developed an adjunct COVID-19 surgical safety checklist and provided guidance on COVID-19 surgical, obstetric, anaesthesia service preparedness, and perioperative provider safety.

Returning to the topic of Lifebox’s latest contribution on surgical headlights, the authors from Ethiopia, Liberia, and the Lifebox Foundation are to be commended for reporting operator feedback on procedures performed and performance of the headlight. The performance issue of headlight dimming during a procedure is one that can be managed for most operations simply by checking battery charge at the beginning, but also having fully charged back-up batteries available. Addressing the issue of wearer comfort, is one that requires practice and getting used to wearing a headlight. There is no doubt that headlights can be uncomfortable if worn too tight, or that they are annoying when too loose and slip downwards off the forehead towards the eyes during a procedure. Headlights also produce heat, so some surgeons don a headband if the combination of the temperature of the theatre, the stress of the surgery and the heat of the headlight generate too much sweat. The argument that surgeons do not want to wear a headlight because they do not find it comfortable is somewhat weak. Repeated use will normalise the wearing of a headlight which, for many procedures, including intra-abdominal surgery, should be regarded as ‘best practice’ rather than something optional. Just over a hundred years ago some surgeons were still arguing about the necessity of wearing gloves. Let us hope we do not have to wait much longer for surgeons everywhere to be asking for a headlight and not leaving them on the side bench because it is not what they are used to.

Those who provided longer-term follow-up (20 out of 64 surgeons) after six months generally reported improved surgical outcomes, more confidence in performing procedures and less hospital-initiated postponements [1]. However, one of the disappointing findings of this mixed methods study was the relatively poor response rate by surgeons who received a free, donated headlight, and, as a group, their limited reporting of the operations they performed. This demonstrates a certain lack of willingness to provide quality control feedback on the headlight performance and its impact. If this reluctance to audit is reflective of their surgical practice in general, their understanding of the safety and quality of their whole practice will be limited. Audit of surgical activity, process, and outcomes should be a standard of care. Patients and the community expect us to reflect on our practice and to be aware of our perioperative outcomes. An additional 45 headlights were distributed to surgeons in Liberia (51 in total), yet only 10 (20%) provided long-term (> 6 months) feedback [1].

A similar problem with inconsistent audit has been reported by SIGN fracture international. Surgical Implant Generation Network (SIGN) is a non-profit organisation that provides free SIGN nails for fracture fixation in return for uploading cases treated into the SIGN online surgical database (SOSD). It provides LMICs with essential items for internal fixation of fractures for no cost, but in return requires users to provide a retrospective upload of use and outcomes to monitor quality and safety. In 2011, SIGN reported outcomes for over 34,000 patients in 55 LMICs with a low infection rate of 0.7% (1.2% in tibial fractures) [5]. It is not only reasonable that donors address safety, quality and outcome issues associated with their products, but also that it should be part of our culture that surgeons and hospitals should proactively audit the structure, process and outcomes of surgical practice. Where this does not occur there can be little quality assurance, and opportunities will be missed for learning and quality improvement.
